# IFN-γ protects the human blood-brain barrier from Toxoplasma gondii-mediated dysfunction via CCL2 suppression

**DOI:** 10.21203/rs.3.rs-8524866/v1

**Published:** 2026-01-14

**Authors:** Mia C Somenzi, Kameron T Bell, Abigail M Bitters, Collin S Stratton, Samantha R Springston, Kamryn E Zadeii, Rylee J Anderson, Daisy Woellner-Santos, Scott G Canfield, Américo H López-Yglesias

**Affiliations:** Indiana University School of Medicine

## Abstract

The obligate intracellular parasite *Toxoplasma gondii* infects nearly one-third of the global population, yet its impact on human blood–brain barrier (BBB) function remains poorly defined. In this study, we use human induced pluripotent stem cell-derived (iPSC) brain-like microvascular endothelial cells (BMECs), a physiologically relevant BBB model, to investigate if *T. gondii* infection directly compromises barrier integrity. We show that infection induces robust monocyte chemotactic protein-1 (also known as CCL2) secretion from BMECs, and that CCL2 itself serves as a previously unrecognized driver of BBB dysfunction. Notably, interferon-gamma (IFN-γ) limits BMEC-derived CCL2 release and protects against parasite-mediated barrier damage. These findings identify an IFN-γ-dependent, BMEC-intrinsic defense mechanism that helps preserve central nervous system (CNS) integrity during infection. Collectively, this work establishes a mechanistic foundation for understanding how *T. gondii* disrupts the human BBB and reveals a novel protective role for IFN-γ in mitigating CNS barrier dysfunction.

## Introduction

The obligate intracellular protozoan parasite *Toxoplasma gondii* is estimated to infect one-third of the global human population. Humans acquire *T*. *gondii* through food, water, or the accidental ingestion of soil that is contaminated with bradyzoites or oocysts.[1] Once ingested, *T*. *gondii* will invade host enterocytes and begin to transform into the parasite’s fast-replicating stage, tachyzoites. In immunocompetent hosts, *T*. *gondii* infections are commonly asymptomatic due to robust CD4^+^ T cell-derived IFN-γ immune responses, resulting in rapid parasite clearance.[2-6] Unfortunately, even in immunocompetent individuals, *T*. *gondii* will evade host-mediated clearance, replicate, and egress from vascular endothelial cells, transforming into slow-growing cysts within neurons leading to chronic infection.[7-11] Ultimately, *T. gondii* breaches the blood-brain barrier (BBB) and establishes a lifelong infection within host neurons. Notably, latent *T. gondii* infection have also been linked to cognitive decline, suggesting a potential association between chronic parasitic infection and progressive deterioration of the BBB over time.[7, 8]

The BBB is comprised of brain microvascular endothelial cells (BMECs) that line brain capillaries, providing a physical, transport, and metabolic barrier between the brain and bloodstream. Compared to non-brain endothelium, BMECs display a much greater restriction to paracellular and transcellular diffusion of ions, small molecules, and proteins by expression of tight junction proteins.[12-15] These localized tight junction proteins result in high trans-endothelial electrical resistance (TEER), representative of a tight barrier.[16-18] The physical barrier properties of the BBB allow for control of nutrient and metabolite flow into and out of the brain with a wide array of molecular transport systems, including nutrient and efflux transporters.[19-21] The BBB is fundamental in protecting the brain parenchyma from harmful circulating factors in the vasculature. Astrocytes, pericytes, neurons, and neural stem cells contribute to the induction and regulation of these barrier properties.[14, 22-26] Despite these biological barriers to prevent entry of pathogens into the brain, *T*. *gondii* nonetheless crosses the BBB in one of three potential pathways: i) Trojan horse- parasitized leukocyte extravasates to the brain parenchyma; ii) Paracellular transmigration- the migration of extracellular tachyzoites via tight junctions; iii) Transcellular migration- active invasion of the endothelium, replication, and basolateral egress into the parenchyma; ultimately infecting neurons and establishing a latent infection for the lifetime of the host.[9-11]

Previous studies using both immortalized and primary cells from canines, rodents, and humans have generated contradictory findings regarding the role *T*. *gondii* in mediating BBB dysfunction. Previously reported results have shown no effect on junctional proteins or barrier function of rodent intestinal cells and murine brain tissue-derived endothelial cells following exposure to the virulent type I RH strain of *T*. *gondii*,[27, 28] whereas multiple studies have shown that tachyzoite (RH)-derived proteases are sufficient to disrupt the barrier integrity of human colon endothelial Caco-2 and canine kidney (MDCK) monolayers *in vitro*.[29, 30] However, the translational aspects of these studies are limited as their findings were in non-human and non-brain barrier models which lack the phenotypic fidelity of the human BBB. Hence, the current literature has evidence from *in vitro* and *in vivo* models with contradictory findings regarding the role of *T. gondii-*mediated barrier disruption, likely due to inconsistent temporal and cellular datasets, translational differences between models, *T*. *gondii* strain (type I vs II), and/or parasite dose.

To bridge these paradoxical findings, we have employed human induced pluripotent stem cell (iPSC)-derived BMECs, which closely mimic the *in vivo* properties of the BBB including robust barrier integrity, expression of localized tight junction proteins, active efflux transporters, and reduced transcellular transport.[25, 31, 32] Strikingly, our findings have revealed that cellular invasion by *T*. *gondii* mediates the loss of BMEC tight junction proteins and their barrier integrity. Mechanistically, we uncovered that IFN-γ protects BMECs from *T. gondii*-induced dysfunction, in part by reducing BMEC-derived monocyte chemotactic protein-1 (MIP1; also known as CCL2). CCL2 is a key chemokine for recruiting inflammatory monocytes, which we demonstrate also directly contributes to the loss of barrier integrity during infection.[33] These findings establish IFN-γ as a critical factor in preserving the human BBB during *T. gondii* infection, mediated in part through CCL2 suppression.

## Materials and Methods

### Differentiation of Brain Microvascular Endothelial Cells

Cells were differentiated as previously published.[34] Briefly, human iPSCs (IMR90, WiCell) were detached and singularized from culture wells using Accutase (Life Technologies) for a 45-minute incubation at 37°C. Post-detachment, the cells were plated at a density of 10,000 cells/cm^2^. The culture medium used was mTESR (STEMCELL Technologies), enhanced with 10 μM ROCK kinase inhibitor (STEMCELL Technologies), in 6-well plates (Fisher Scientific) that had been pre-coated with Matrigel (Fisher Scientific). These conditions were maintained for 3 days at 37°C, with daily replenishment of mTESR. Differentiation was initiated by switching to unconditioned medium (UM), composed of Dulbecco’s Modified Eagle’s Medium/Ham’s F12 (DMEM-F12, Fisher Scientific), 20% Knockout Serum Replacement (Fisher Scientific), 1 mM GlutaMAX (Life Technologies), 0.1 mM β-mercaptoethanol (Sigma Aldrich), and MEM Non-Essential Amino Acids (Life Technologies), which was refreshed daily for 6 days. Following this period, the medium was substituted with Endothelial Cell (EC) medium, specifically EC (+/+) medium consisting of Human Endothelial Serum-Free Medium (hESFM, Life Technologies) supplemented with 1% B27 (Fisher Scientific), 20 ng/mL basic fibroblast growth factor (bFGF, STEMCELL Technologies), and 10 μM retinoic acid (RA; Sigma Aldrich). After 2 days in EC (+/+) medium, cells were singularized once more, and re-plated on substrates pre-coated with Matrigel (Corning Life Sciences) on plates and coverslips. For final seeding, BMECs were allocated at specific densities suited to various assays: 1.12 cm^2^ inserts in a Transwell© filter system (Fisher Scientific), 24-well plates and 96-well plates (Fisher Scientific) at densities of 10^6^ cells/cm^2^, 125,000 cells/cm^2^, and 300,000 cells/cm^2^ respectively. 24 h after seeding, the media was replaced with EC medium supplemented with 1% B27 and no bFGF (EC +/−).

#### Toxoplasma gondii infections

*T*. *gondii* tachyzoites were maintained through serial passage in Hs27 fibroblasts (RL-1634; ATCC). Hs27 cells were infected with Prugniaud (Pru) *T*. *gondii* strains at multiplicity of infection (MOI) of 0.1:1, 1:1, or 6:1 for the indicated times. In some experiments, BMECs were stimulated with 5 ng/mL of recombinant human IFN-γ (rIFN-γ; BioLegend) solution for 24 h prior to *T*. *gondii* infection. In some experiments, BMECs were treated with 100 μg/mL of anti-CCL2 (BioxCell) and infected immediately after. In some experiments, BMECs were stimulated with 40 μg/mL of recombinant human CCL2 (rCCL2; BioxCell) and were not infected.

### Trans-Endothelial Electrical Resistance

TEER was assessed using an EVOM ohmmeter equipped with STX2 electrodes (World Precision Instruments). Measurements of TEER in BMECs were taken at the time of exposure to *T. gondii* tachyzoites 3 days after purification. Subsequent TEER measurements were conducted at intervals of 3, 6, 12, 24, 48, 72, and 96 h after exposure. To correct for the surface area of the inserts, the recorded values were adjusted by subtracting the resistance values from a blank, non-cell seeded Transwell, and then multiplying by the area of 1.12 cm^2^. The results of TEER are expressed in units of ohm-centimeters squared (Ω x cm^2^).

### Immunofluorescence and Area Fraction Index/Discontinuous Junction %

24 h after infection with *T*. *gondii*, BMECs were subjected to three washes using Phosphate Buffered Saline (PBS; Sigma Aldrich). Subsequently, cells underwent fixation with cold methanol (100%; Sigma Aldrich) for 15 minutes. Post-fixation, cells were treated with a blocking solution consisting of PBS mixed with 10% goat serum (Sigma Aldrich) and incubated for 1 h at room temperature (RT) on a rocker. After three PBS washes, cells were incubated with primary antibody that was diluted in the blocking solution.[32] This incubation occurred overnight at 4°C on a rocking platform. Following another three PBS washes, secondary antibodies diluted 1:200 in the blocking solution, were applied. Cells were then left to incubate with these secondary antibodies for 1 h at RT in darkness on a rocking platform. After this, cells were washed three times with PBS and incubated with 4',6-diamidino-2-phenylindole (DAPI), diluted 1:1000 in blocking solution, for 15 min at RT in the dark on a rocking platform. Following DAPI staining and three subsequent PBS washes, images were captured using an Olympus PROVIS AX70 motorized fluorescent microscope (Olympus) equipped with a SPOT Pursuit USB camera (SPOT Imaging).

Post-imaging analysis involved quantification of discontinuous tight junction proteins (claudin-5, occludin, and zonula occluden-1 (ZO-1)) using Image J, analyzing at least 10 fields containing roughly 80 cells per field from three separate differentiations. The area fraction index was calculated to assess the area of each image that showed immunoreactivity for claudin-5, occludin, or ZO-1.

### Western Blot

After a 24 h exposure to *T*. *gondii*, BMECs were subjected to a triple wash with PBS. BMECs were then lysed using cold Pierce^™^ RIPA buffer, which included Halt^™^ Protease and Phosphatase Inhibitor (ThermoFisher), on a rocker at 4°C for a period of 30 minutes. Lysates were collected post-centrifugation at refrigerated conditions. Total protein content of the lysates was determined following the protocol of the Pierce^™^ Rapid Gold BCA Protein assay kit (ThermoFisher), with absorbance readings taken at 480nm on a Synergy HTX Multi-Mode reader. Proteins, precisely 10 mg, were loaded onto either 12% or 4–15% gradient precast polyacrylamide gels (Bio-Rad) for electrophoresis. A Bio-Rad Mini-Protean^®^ Tetra Vertical Electrophoresis Cell (Bio-Rad) was utilized for gel separation in SDS (Tris/Glycine/SDS buffer), at 120 volts for an hour for 12% gels and 70 minutes for the 4–15% gradient gels. Following this, proteins were translocated to Immuno-Blot^®^ PVDF membranes (Bio-Rad) and subjected to a transfer process in a Tris/Glycine buffer containing 20% methanol for 1 h at 100 volts. The membranes, once transferred, were immersed in a blocking solution of Tris-buffered saline with 0.1% Tween 20 (TBST) and 5% non-fat dry milk for 1 h at ambient temperature, succeeding a wash with TBST. After three TBST washes, membranes were incubated with primary antibodies specific to tight junction proteins (claudin-5, occludin, ZO-1) and β-actin, as listed in the blocking solution overnight at 4°C. After primary antibody application, membranes were thoroughly rinsed for a quarter of 1 h each to eliminate unattached primary antibodies.

For the detection phase, membranes were incubated with HRP-conjugated secondary antibodies (ThermoFisher) in TBST mixed with 5% non-fat dry milk for 60 minutes at RT. Following three 15-minute washes, membrane imaging was performed using a LI-COR C-DiGit^®^ Blot Scanner (LI-COR). Image quantification was executed using Image J software version 1.52, with normalization of the data against β-actin levels.

### Visualization of BMEC Death

Following 24 h *T. gondii* infection, supernatants were decanted from BMECs. BMECs were washed with 100 mLs of PBS. 100 mLs of propidium iodide (PI) staining solution (Millipore Sigma) was added to each sample. 100 mLs of DMSO was added to a well acting as a positive control. All samples were incubated in the dark for 5 minutes. Cells were visualized microscopically and quantified via flow cytometry.

### Sodium Fluorescein Permeability Measurements

The permeability of the barrier was determined by utilizing sodium fluorescein (NaF; 10 μM, 376 Daltons; Sigma Aldrich) applied to BMECs cultured on transwell inserts. The BMECs were subjected to a 24 h exposure to *T*. *gondii*. Post-exposure, the media was replaced and pre-warmed EC +/− media containing NaF was introduced to the upper compartment of each Transwell. Concurrently, EC +/− media without the fluorescent dye was added to the lower chamber. The cells, along with the NaF, were then incubated at 37°C on a rotating platform. Sampling was conducted every 15 minutes from the lower chamber of each Transwell for 1 h. At each sampling point, 150 μL of the solution was transferred to a black 96-well plate (Fisher Scientific), and an equal volume of fresh, pre-warmed EC +/− media was replenished in the lower chamber of the Transwell. The samples were then quantitatively measured for fluorescence using a Synergy HTX Multi-Mode reader (BioTek), and the permeability coefficients were deduced from the clearance rate of NaF.

### Cytokine measurements

The concentrations of cytokines IL-6, IL-8, CCL2, and IFN-γ from *T. gondii*-infected experimental cell supernatants were analyzed using LEGENDplex Human Inflammation Panel 1 (13-plex) according to manufacturer’s instructions (BioLegend).

### Statistical analysis

Each experimental group consisted of iPSC-derived BMECs from at least three separate differentiations. For the statistical analyses, Prism (Version 10; GraphPad, La Jolla, CA) was used. Statistical significance determined via one-way analysis of variance (ANOVA) with post-Tukey test. These data were considered statistically significant when *p* values were < 0.05. Error bars on all figures are representative of the standard error mean (SEM)

## Results

### T. gondii infection mediates reduced barrier resistance of human iPSC-derived BMECs.

Establishment of chronic *T. gondii* infection in human neurons requires penetration of the BBB. Therefore, we hypothesize that *T. gondii* infection mediates BBB dysfunction prior to chronic *T. gondii* infection in human neurons. To test this hypothesis, we utilized human iPSC-derived BMECs that mimic several *in vivo* properties of the BBB. These properties include robust barrier integrity, expression of localized tight junction proteins, active efflux transporters, and reduced transcellular transport.[25, 31, 32]

**Figure 1:**
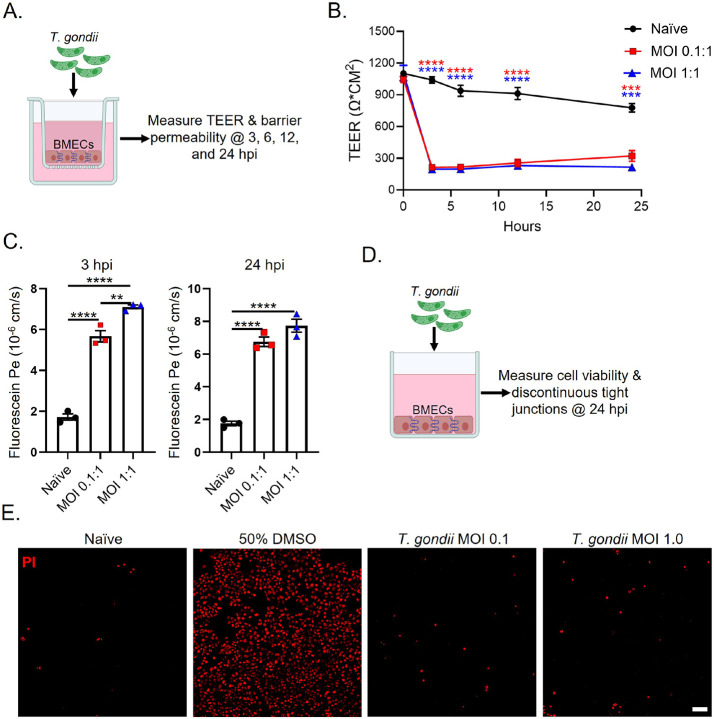
*T. gondii* infection mediates reduced barrier resistance of human iPSC-derived BMECs. A) Schematic model of the experimental design with *T. gondii* and BMECs to measure the maximum TEER. B) Maximum TEER values were measured following infection with *T. gondii* at 3, 6, 12, & 24 hpi C) Barrier permeability was assessed via NaF following infection with *T. gondii* at 3 & 24 hpi. Permeability coefficients were calculated based on cleared volume of NaF from top chamber vs. bottom chamber. D) Schematic model of the experimental design with *T. gondii* and BMECs to measure discontinuous tight junctions by IF and cell viability. E) IF staining with propidium iodide (PI) to assess cell viability 24 hpi. Data represents mean values ± SEM from at least three independent differentiation experiments. Statistics: One-way ANOVA with post-Tukey test, ** P<0.01, *** P<0.001, **** P< 0.0001.

To determine the effect of *T. gondii* infection on barrier tightness, TEER was measured immediately following parasitic infection of BMECs with MOIs of 0.1:1 and 1:1 at 3, 6, 12, and 24 hours post-infection (hpi) (Fig. 1A). Infection significantly reduced barrier integrity as early as 3 hpi continuing for 24 h (Fig. 1B). Next, we evaluated paracellular NaF permeability 24 hpi. The results demonstrate at either an MOI of 0.1:1 or 1:1 that *T. gondii* infection mediates a significant increase in barrier permeability (Fig. 1C) that correlates with decreased barrier integrity.

Our findings reveal that *T. gondii* infection mediates significant reduction in barrier integrity and increase in barrier permeability of the BBB (Fig. 1B, C). Yet, it is important to note that following several rounds of replication the parasite will egress from the host cell resulting in the host cell’s death.[35] Thus, we next examined if the loss of barrier integrity and increased barrier permeability was related to *T. gondii-* mediated cellular death. To test for cell death following parasitic infection, we stained BMECs with PI, visualized PI using immunofluorescence (IF) and flow cytometry, and measured LDH release. However, we found no difference between naïve and infected BMECs 24 hpi (Fig. 1D, **data not shown**). These findings indicate that acute *T. gondii* infection compromises the integrity of the human BMEC barrier independently of BMEC cell death.

#### Human iPSC-derived BMECs lose tight junction localization and expression during T. gondii infection

**Figure 2:**
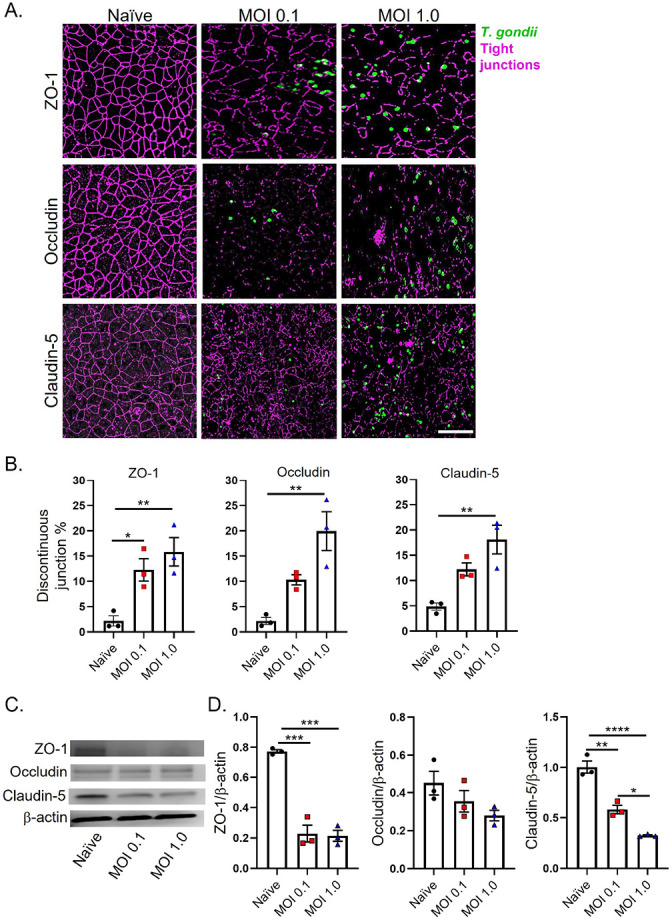
*T. gondii* infection disrupts tight junction localization and expression in human iPSC-derived BMECs. Tight junction integrity was assessed in human iPSC-derived BMECs following 24 h infection with *T. gondii* at MOls of 0.1:1 and 1:1. A) IF staining of tight junction proteins ZO-1, occludin, and claudin-5 in uninfected BMECs and BMECs infected with *T. gondii*. B) Quantification of tight junction disruption was performed by calculating the area fraction index of ZO-1, occludin, and claudin-5 immunoreactivity. C) Western blot analysis of ZO-1, occludin, and claudin-5 protein levels in BMECs 24 hpi with β-actin used as a loading control. D) Densitometric quantification of protein expression normalized to β-actin using ImageJ software. Data represents mean values ± SEM from at least three independent differentiation experiments. Statistical significance determined via one-way ANOVA with post-Tukey. * P<0.05 ** P<0.01 *** P<0.001 **** P< 0.0001.

We next investigated changes in tight junction protein localization and expression in *T. gondii-*infected BMECs. To determine the effect of *T. gondii* infection on tight junction localization we performed IF of BMECs 24 hpi. Tight junction staining revealed a dramatic loss of ZO-1, occludin, and claudin-5 localization with either a MOI of 0.1:1 or 1:1 at 24 hpi (Fig. 2A). Notably, the average percent disruption of all tight junction proteins increased with a positive correlation to the MOI (Fig. 2B).

We next performed Western blot analysis of tight junction proteins ZO-1, occludin, and claudin-5 to determine their expression levels. BMECs were infected with MOIs of 0.1:1 or 1:1 and protein levels were assessed 24 hpi. These results demonstrate that *T. gondii* infection of BMECs results in the dramatic loss of ZO-1, occludin, and claudin-5 expression (Fig. 2C, D).

### IFN-g protects the BBB from T. gondii-mediated dysfunction.

**Figure 3:**
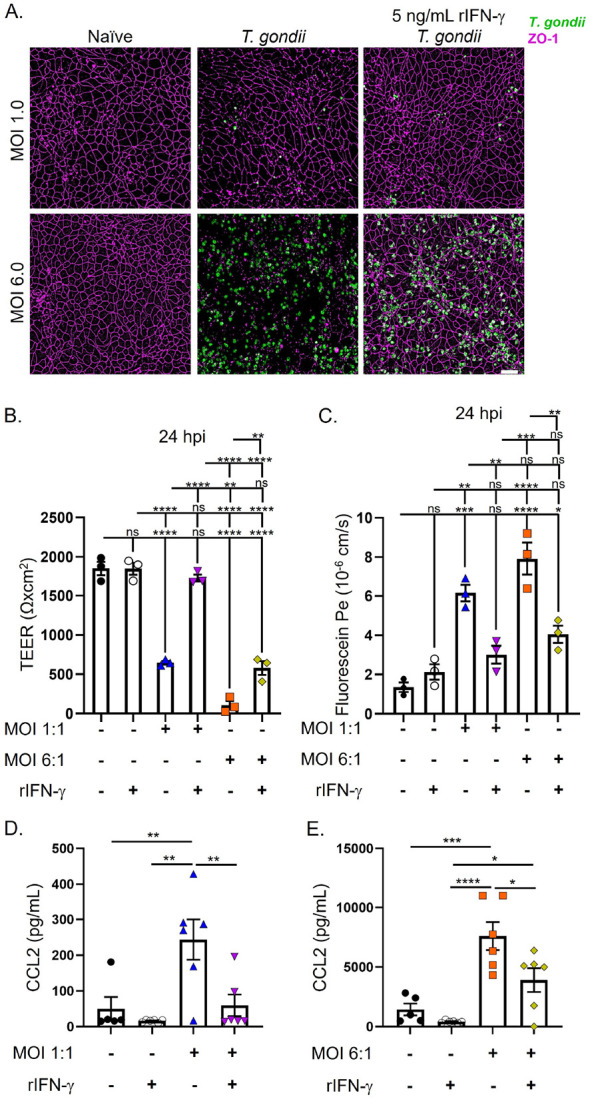
IFN-gprotects the BBB from *T. gondii*-mediated breakdown. Barrier integrity was assessed in human iPSC-derived BMECs stimulated with or without IFN-g 24 h prior to being infected with *T. gondii* at MOls of 1:1 or 6:1 for 24 h. A) ZO-1 localization on BMECs 24 h following parasite infection. B) Maximum TEER values were measured following IFN-g stimulation and *T. gondii* infection at 24 hpi. C) NaF permeability was assessed following IFN-g stimulation and *T. gondii* infection at 24 hpi. Permeability coefficients were calculated based on cleared volume of NaF from top chamber vs. bottom chamber. D, E) CCL2 analysis by flow cytometry-based multiplex of BMEC-derived supernatants following IFN-g stimulation and *T. gondii* 24 hpi. Data represents mean values ± SEM from at least three independent differentiation experiments. Statistical significance determined via one-way ANOVA with post-Tukey. * P<0.05 ** P<0.01 *** P<0.001 **** P< 0.0001.

The cytokine IFN-γ is essential for host resistance to *T. gondii*; however, our group and others have shown that IFN-γ contributes to intestinal immunopathology and BBB permeability during parasitic, bacterial, and viral infections.[36-38] Therefore, we hypothesized that IFN-γ stimulation would impair BMEC barrier integrity and disrupt tight junctions. Strikingly, we observed that IFN-γ did not result in tight junction loss, nor did it compromise barrier integrity in human BMECs (Fig. 3A-C). Notably, we observed that IFN-γ treatment was sufficient to significantly inhibit parasite-mediated tight junction loss and barrier dysfunction, even when infected with a high dose (MOI 6:1) of *T. gondii* (Fig. 3A). Additionally, we observed that pre-treatment of BMECs with IFN-γ resulted in no loss of barrier permeability by 24 hpi (Fig. 3B, C). These data indicate that IFN-γ alone does not damage human BMEC integrity and demonstrates that IFN-γ plays a critical role in maintaining a functional BBB during parasitic infection.

Next, we aimed to determine the other molecular factors potentially contributing to *T. gondii*-mediated BBB dysfunction. It is well-established that *T. gondii* infection induces the production of the pro-inflammatory cytokine TNF, which has been shown to play a protective role in host resistance to infection.[39] Yet, it has also been demonstrated that TNF can trigger a “leaky” BBB.[40] We therefore predicted that *T. gondii* infection would induce significant expression of BMEC-derived TNF. To test our prediction, BMECs were infected with *T. gondii* and supernatants were collected 24 hpi for cytokine analysis via a flow cytometry-based multiplex. The cytokine multiplex allows for simultaneous quantification of 13 human inflammatory cytokines. From the 13 cytokines measured from infected human iPSC-derived BMECs, our data shows that *T. gondii* significantly upregulated IL-6, IL-8, and CCL2 production 24 hpi with an MOI of 1:1 or 6:1 (Fig. 3D, E, **data not shown**). Markedly, we also observed that treating BMECs with rIFN-γ prior to infection significantly reduced CCL2 production 24 hpi, but did not affect IL-6 or IL-8 cytokine levels (Fig. 3D, E, **data not shown**). These data suggest that varying MOIs can increase BMEC-derived CCL2 while IFN-γ can significantly impede its production during infection.

### T. gondii -mediated BMEC-derived CCL2 significantly contributes to human BBB dysfunction.

**Figure 4:**
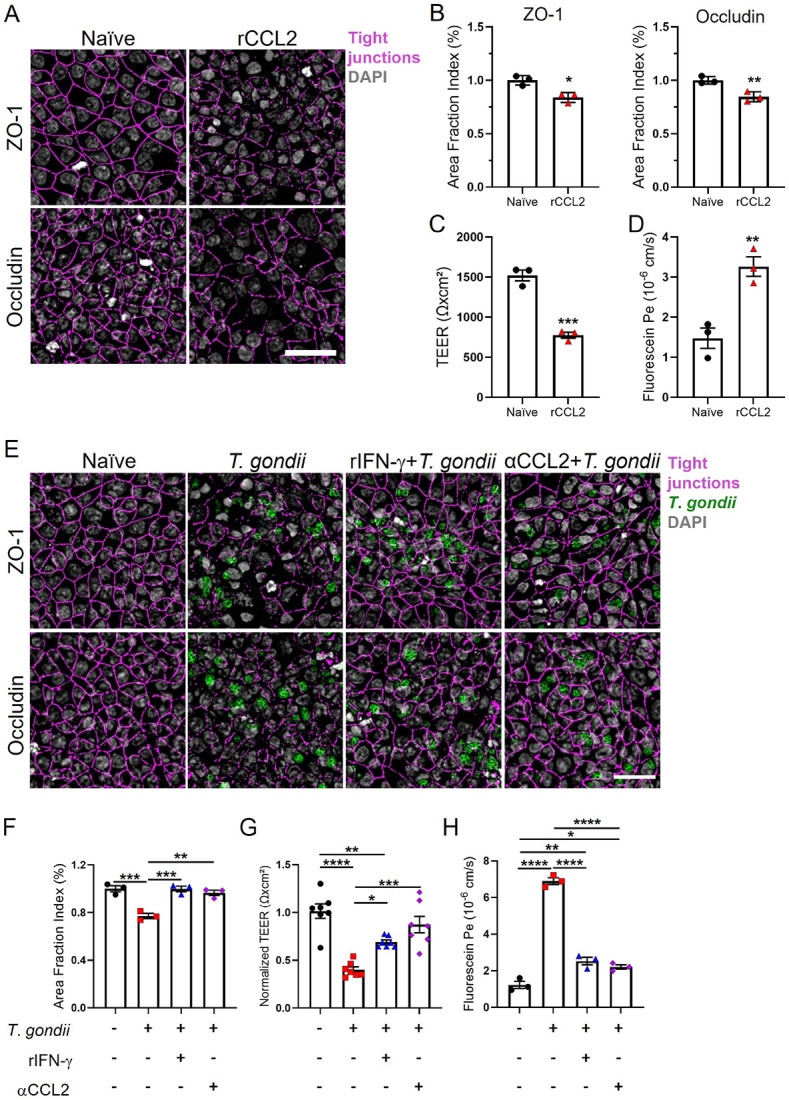
IFN-gprotects the human BBB from *T. gondii*-induced dysfunction by suppressing CCL2 A) IF staining of tight junction proteins ZO-1 and occludin in human iPSC-derived BMECs under control conditions and following 24 h stimulation with rCCL2. B) Quantification of ZO-1 and occludin localization using area fraction index analysis. C) TEER measurements taken 24 h after rCCL2 stimulation to assess barrier integrity. D) NaF permeability assay performed 24 h post-rCCL2 stimulation. Permeability coefficients were calculated based on the cleared volume of NaF from the top to bottom chamber. E) IF staining of ZO-1 and occludin in BMECs only, BMECs stimulated with rIFN-g and then infected with *T. gondii*, or BMECs treated with anti-CCL2 prior to being infected with *T. gondii* for 24 h. F) Quantification of ZO-1 localization using area fraction index. G) TEER measurements from BMECs under the same conditions as in panel E. H) NaF permeability assay performed under the same conditions as in panel E, with permeability coefficients calculated as described in panel D. Data represents mean values ± SEM from at least three independent differentiation experiments. Statistical significance determined via one-way ANOVA with post-Tukey. * P<0.05 ** P<0.01 *** P<0.001 **** P<0.0001.

We next wanted to determine the role of CCL2 in mediating BBB damage. To determine if CCL2 alone could mediate BMEC dysfunction, we treated BMECs with rCCL2 for 24 h and then assessed tight junctions and barrier permeability. Our data indicates that CCL2 is sufficient to cause significant tight junction disruption (Fig. 4A), quantified via an area fraction index of the tight junction confluency (Fig. 4B). Moreover, barrier integrity significantly declined, as indicated by reduced TEER (Fig. 4C), and barrier permeability significantly increased, as quantified by NaF flux (Fig. 4D). These results demonstrate that CCL2 is sufficient to mediate BBB dysfunction even in the absence of microbial infection.

**Figure 5:**
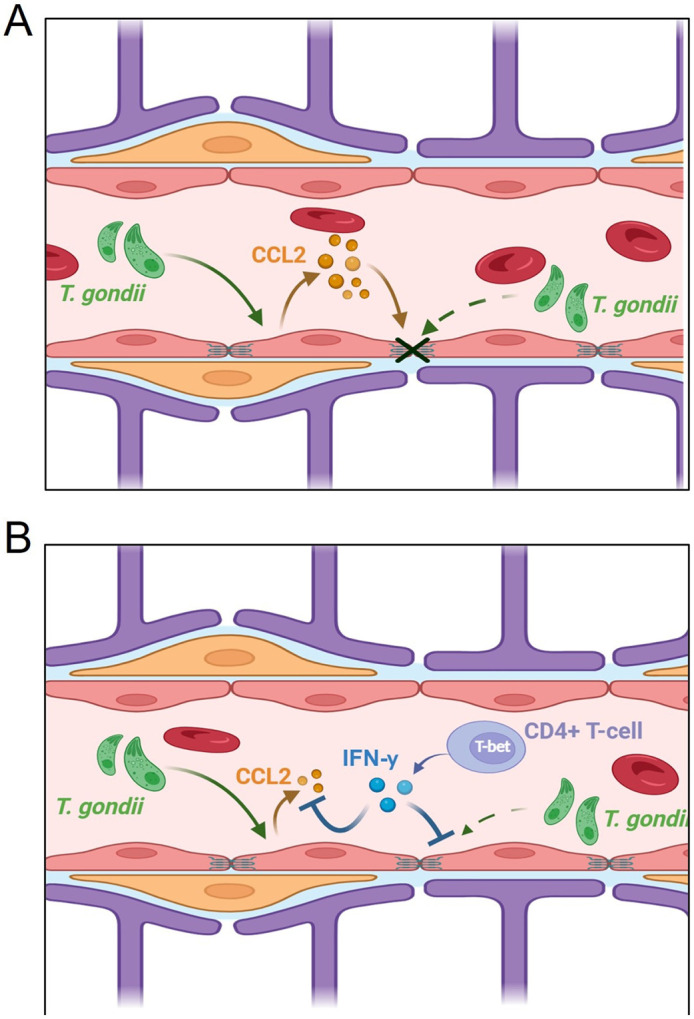
IFN-gprotects the BBB from *T. gondii*-mediated breakdown by preventing the production of BMEC-derived CCL2 Schematic model of the factors mediating BBB breakdown and the cytokinetic interplay. A) *T. gondii* infection in BMECs leads to production and auto-reception of CCL2, exacerbating *T. gondii-* mediated barrier disruption. B) IFN-g blocks the production of CCL2, preserving barrier integrity during *T. gondii* infection.

As we have previously shown, an important effect of IFN-γ stimulation of BMECs is the reduced production of CCL2 during infection. Therefore, we next assessed whether CCL2 directly contributed to BBB dysfunction during infection. Treatment with anti-CCL2 prevented the loss of tight junctions 24 hpi (Fig. 4E, F). This tight junction preservation is coupled with increased barrier integrity and decreased barrier permeability as quantified by TEER and NaF, respectively (Fig. 4G, H), that is comparable to IFN-γ treated BMECs. Collectively, these results demonstrate that *T. gondii* infection triggers BMEC-derived CCL2 production, which significantly contributes to BBB dysfunction, whereas IFN-γ preserves barrier integrity by suppressing *T. gondii*-induced CCL2 expression in BMECs (Fig. 5).

## Discussion

To our knowledge, this is the first study using human iPSC-derived BMECs to demonstrate that the obligate intracellular neurotropic parasite, *T*. *gondii*, mediates BBB dysfunction during acute infection. We show that *T. gondii* directly induces CCL2 release from BMECs, and that CCL2 contributes to barrier damage. Moreover, our data reveals that IFN-γ, the type II interferon critical for leukocyte activation and parasite clearance, contributes to maintaining BBB integrity by significantly limiting BMEC-derived CCL2 release during infection. Collectively, these findings underscore the potential long-term impact of parasite-mediated immunopathology that is estimated to affect nearly one-third of the global population. Notably, we have demonstrated the detrimental effects of *T. gondii* on a relevant human BBB model and have identified a novel protective role for IFN-γ in preserving CNS integrity.

Previous studies using both immortalized and primary cells from canines, rodents, and humans have generated contradictory findings regarding the role *T*. *gondii* in mediating BBB dysfunction. However, the translational aspects of these studies are limited as their findings were in non-human and non-brain barrier models which lack the phenotypic fidelity of the human BBB.[28-30] Therefore, to determine if *T*. *gondii* mediates damage to the human BBB, we utilized human iPSC-derived BMECs, which closely mimic the *in vivo* properties of the BBB including robust barrier integrity, expression of localized tight junction proteins, active efflux transporters, and reduced transcellular transport.[34] These critical barrier properties protect the brain parenchyma from unwanted molecules reaching the vulnerable neuronal cell types. Furthermore, we employed a type II parasite strain, due to is epidemiological relevance of being the most prevalent form of *T*. *gondii* in North America and Europe, and its ability to recapitulate long-term latent infections in rodent models. While our study demonstrates that type II *T*. *gondii* tachyzoites rapidly induce barrier damage, additional work is required to resolve if all endothelial cells are susceptible to parasite-mediated dysfunction and if this damage is limited to certain *T*. *gondii* strains.

*T*. *gondii* infection triggers a rapid CD4^+^ T-helper 1 (Th1)-mediated immune response, which is classically defined by lymphocyte-derived IFN-γ production that is indispensable for protective immunity.[2, 41-44] The majority of research on IFN-γ has focused on its ability to eliminate *T*. *gondii* from infected cells and stimulate host defense. However, recent work by Yarovinsky et al. shows that during *T*. *gondii* infection, IFN-γ mediates intestinal immunopathology and that its signaling in Paneth cells results in their death. [36, 45] Multiple groups have also shown that IFN-γ mediates BBB permeability in the context of both bacterial and viral CNS infection.[37, 38] Moreover, IFN-γ drives the expression of chemokine CXCL10 to enhance leukocyte recruitment to the BBB and exacerbate barrier dysfunction.[46, 47] Notably, our results demonstrate that IFN-γ significantly protects human iPSC-derived BMECs from *T*. *gondii*-mediated barrier dysfunction. These findings suggest that IFN-γ signaling in BMECs can protect glial cells from microbial-induced damage or death, indicating an IFN-γ-dependent, BMEC-mediated innate defense mechanism that limits neuronal infection and maintains barrier integrity.

The chemokine CCL2 is critical for the recruitment of inflammatory monocytes which express high levels of CCR2. During *T*. *gondii* infection CCL2 recruits CCR2 + inflammatory monocytes from the bone marrow that migrate into infected tissues, which is critical for host resistance, as CCR2- or CCL2- deficient mice have been shown to rapidly succumb to *T. gondii* infection.[48-50] In the brain, microglia and brain-infiltrating myeloid are the primary source of CCL2 during acute infection.[51] Meanwhile, during chronic *T. gondii* infection, astrocytes account for 75% of the CCL2-producing cells.[52] Collectively, these studies indicate that during *T. gondii* infection there is continuous CCL2 production in the brain that plays a significant role in controlling parasite replication by recruiting myeloid cells.[53] Moreover, a recent study by Garcia et al. aligns with our own results, reporting that human umbilical vein endothelial cells (HUVECs) exhibit a pro-inflammatory transcriptional profile—marked by increased IL-6, IL-8, and CCL2 expression—following *T. gondii* infection.[54] Previous studies have also demonstrated that CCL2 can promote vascular permeability in a rodent lung metastasis model.[55] Based on this, we investigated whether a similar effect occurs in our *in vitro* BBB system. Strikingly, our findings reveal a previously unrecognized role for CCL2 in driving barrier dysfunction within the CNS during infection.

Our findings uncover a previously unrecognized mechanism in which IFN-g signaling in iPSC-derived BMECs protects against *T*. *gondii*-induced barrier dysfunction, partly through IFN-γ-mediated suppression of BMEC-derived CCL2. These results highlight a potential long-term CCL2-dependent immunopathology of the BBB that may persist throughout the lifetime of a chronically infected host. Notably, a meta-analysis of eight studies found that people with *T*. *gondii* infection had a 1.53 times higher risk of developing Alzheimer’s disease (AD).[56] Taken together, our study suggests a possible link between *T*. *gondii*-mediated BBB damage and heightened susceptibility to AD and cognitive decline.
